# Fluoroscopic-Guided Bilateral Superior Hypogastric Plexus Neurolysis in the Treatment of Intractable Neoplasm-Related Penile Pain

**DOI:** 10.7759/cureus.19991

**Published:** 2021-11-29

**Authors:** Kristy Fisher, Janet Daoud, Christian Gonzalez, Jessica Reyes, MD, Daniel Lopez, Oleg Desyatnikov

**Affiliations:** 1 Psychiatry, HCA Healthcare - Aventura Hospital Center, Aventura, USA; 2 Allopathic Medicine, Nova Southeastern University, Dr. Kiran C. Patel College of Allopathic Medicine, Davie, USA; 3 Pain Management, Spine and Wellness Centers of America, Miami, USA; 4 Anesthesiology, Kendall Regional Medical Center, Miami, USA; 5 Pain Medicine, Kendall Regional Medical Center, Miami, USA

**Keywords:** urology, fluoroscopic, neoplasm-related pain, bilateral superior hypogastric plexus neurolysis, penile pain

## Abstract

Intractable penile pain can be a very difficult condition to address. Studies have shown that both locally advanced and metastatic penile cancer, along with its associated management options and subsequent complications, yield a very poor prognosis, with pain being the most feared symptom. Furthermore, a lack of palliative therapy has been demonstrated in this patient population, with an emphasis on the need for implementing future options. This case depicts a 67-year-old male, with a past medical history of metastatic prostate cancer involving the penis, who presented with intractable penile pain. To the authors’ knowledge, this will be the first documented case of the successful utilization of a bilateral superior hypogastric plexus neurolysis in the management of intractable neoplasm-related penile pain attributed to both radiation-induced injury in the treatment of malignant neoplasm and penile pain secondary to metastatic prostate cancer to the penis. As a currently under-utilized treatment option in the management of intractable neoplasm-related penile pain, this case presentation acts to increase awareness of its potential use, therefore reducing the need for analgesics and the associated burdens, as well as improving patient palliation. Furthermore, this case offers evidence supporting the encouragement of its use in the general management of intractable penile pain due to other pathophysiology.

## Introduction

Intractable penile pain can be a very difficult condition to address, with a multitude of physical and psychological contributing factors, including intimacy of location and sensitivity of its expected and necessary functions. Many medical conditions and procedures can lead to penile pain, including, but not limited to, local diseases/cancer, metastasis, referred pain, neuropathic pain, psychological/psychiatric disorders, penile pain syndrome, and radiation therapy [1]. Studies have shown that both locally advanced and metastatic penile cancer, along with its associated management options and subsequent complications, yield a very poor prognosis, with pain being the most feared symptom. Furthermore, a lack of palliative therapy has been demonstrated in this patient population, with an emphasis on the need for implementing future options [2-6]. To the authors’ knowledge, this will be the first documented case of the successful utilization of a bilateral superior hypogastric plexus neurolysis in the management of intractable neoplasm-related penile pain attributed to both radiation-induced injury in the treatment of malignant neoplasm and penile pain secondary to metastatic prostate cancer to the penis.

## Case presentation

The patient is a 67-year-old male, with a past medical history of diabetes mellitus type II and stage IV metastatic prostate cancer involving the lung, liver, bladder, rectum, and penis, who presented with a chief complaint of painful penile abrasions and dysuria after receiving radiation therapy five days prior. The patient reported a previous diagnosis of prostate cancer 2.5 years ago and had since undergone transurethral resection of the prostate (TURP), elective loop colostomy, and suprapubic catheter placement, along with chemo, radiation, and hormonal therapy. His home medications included gabapentin, metformin, mirtazapine, and simvastatin. 

**Figure 1:**
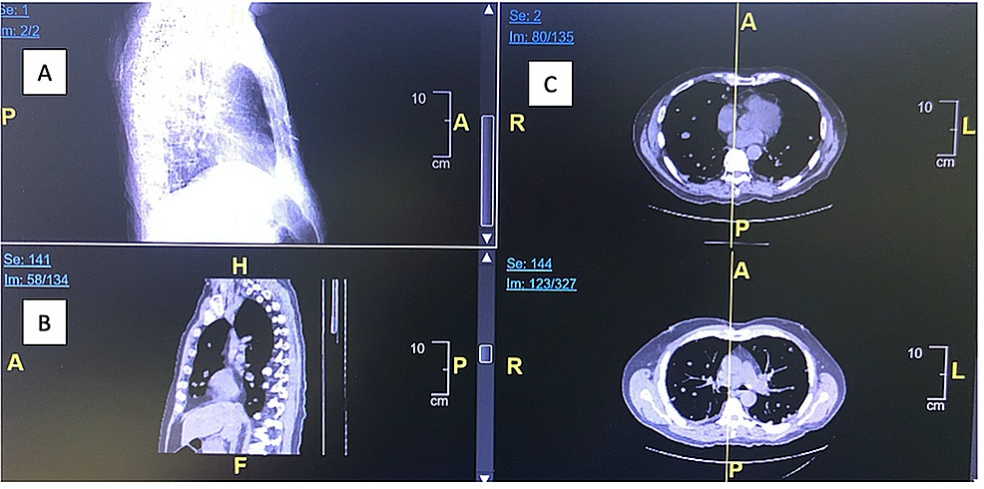
(A-C) CT imaging indicating diffuse metastatic disease involving the liver and lungs, diffuse moderate bulky peritoneal adenopathy along the mesenteric root and liver hilar region, and a small amount of free intraperitoneal fluid.

Upon evaluation, the patient was hemodynamically stable and the physical exam was unremarkable aside from minimal ankle edema, limited range of motion of the extremities due to pain, and skin breakdown on the penis. Laboratory values were unremarkable. CT scan indicated diffuse metastatic disease involving the liver and lungs, diffuse moderate bulky peritoneal adenopathy along the mesenteric root and liver hilar region, and a small amount of free intraperitoneal fluid (Figures 1A-1C).

The patient was admitted to medicine for further evaluation and management.

Upon urologic evaluation, a poor prognosis was determined due to the stage, spread, and exhausted, unsuccessful procedures, with a six-month life expectancy, and further interventions were not indicated. Recommendations of supportive care and hospice placement were made, with consultations for pain management, wound care, and palliative medicine placed. As per pain management, morphine 2mg IV Q4hr prn was initiated and a fluoroscopic-guided neurolytic ganglion plexus block via bilateral superior hypogastric plexus neurolysis was recommended. The procedure was performed in the outpatient setting two days after hospital discharge. The patient reported complete resolution of pain. A follow-up appointment two weeks later revealed persistence in complete pain alleviation. The patient was then lost to follow-up for unknown reasons.

Procedure (fluoroscopic-guided bilateral superior hypogastric plexus neurolysis)

After the risks and benefits were explained, informed consent was obtained. The patient was then taken to the procedure room and positioned prone on the procedure table. The region overlying the right side of the L5 vertebral body was identified using fluoroscopy. The skin was prepped and draped in the usual sterile fashion. The skin and soft tissues were anesthetized using 1% lidocaine. The fluoroscopic beam was directed in a far lateral oblique position until the tip of the transverse process was noted to lie just lateral to the lateral border of the L5 vertebral body. The beam was then tilted so that the transverse process was noted to lie superior to the midpoint of the vertebral body. Using intermittent fluoroscopic guidance, a 3.5-inch 22-gauge needle was then advanced until contact was made with the lateral aspect of the L5 vertebral body at the superior 1/3. At this point, the needle was advanced anteriorly along the vertebral body, while maintaining contact at all times. Needle placement was confirmed using anterior, oblique, and lateral views. When the needle tip was noted to lie at the anterolateral aspect of the L5 vertebral body, needle placement was then confirmed with the injection of iohexol 180. Cephalad and caudad contrast flow was noted along the anterior aspect of the vertebral column. No vascular uptake was noted. No lateral spread was observed. Next, 5mL of 0.25% of bupivacaine + 10mL dehydrated alcohol 97% was injected without resistance after negative aspiration. The same procedure was performed on the left side of the L5 vertebral body. The procedure was performed without any complications.

## Discussion

Intractable penile pain can be quite burdensome not only for the patient but also for the physician managing this difficult and sensitive condition. This case presentation focuses on one particular management option, the fluoroscopic-guided bilateral superior hypogastric plexus neurolysis, particularly in the treatment of penile pain associated with metastasis and complications status-post radiation treatment. However, authors wish to not only raise awareness of the use of such a procedure in the management of this particular condition but also to demonstrate its usefulness in intractable penile pain attributed to various pathophysiology.

Current guidelines for the treatment of pain typically suggest following the World Health Organization (WHO) analgesic ladder. This ladder consists of three steps, which progress in accordance with pain severity for the targeted management of alleviation. The first step typically includes non-opioids, such as acetaminophen. The second step includes mild opioids, such as codeine. For severe and/or persistent pain, despite previous efforts, the third step encompasses the use of strong opioids, such as morphine or fentanyl. Adjuvants, such as antidepressants, anticonvulsants, corticosteroids, and anxiolytics can be implemented throughout each step. Intractable cancer pain resistance to the WHO analgesic ladder affects 10%-15% of cancer patients and was one of the contributing factors responsible for the revision of the analgesic ladder to incorporate the fourth step of non-pharmacological methods. This fourth step is implemented when the analgesic ladder therapy fails to alleviate the pain and/or produces dangerous side effects. At this point, invasive procedures and therapy can be used, including nerve blocks, neurolysis, epidural analgesia, ablative procedures, cementoplasty, as well as palliation radiotherapy [4-8]. In this case presentation, the WHO analgesic ladder was followed in a progressive fashion until the more invasive option of neurolysis was considered.

Nerve blocks are procedures that cause interruption of transmissive signals along a nerve in order to disrupt and halt pain sensation. There are typically two types of nerve blocks, local anesthetic injection or via neuro-destruction. Neuro-destructive methods are typically indicated in the case of intractable chronic pain. An example of a neuro-destructive method is a neurolytic block when degeneration of the nerve fibers is induced via temperature increase (burning), temperature decrease (freezing), or chemicals, such as phenol and alcohol [9,10]. A neurectomy is the complete excision of a nerve to permanently remove that nerve section, but due to regeneration of the nerve and its signal, this procedure is not common practice [11,12].

Literature indicates many different interventional procedures in managing cancer-associated pelvic and perineal visceral pain. Many of these procedures include nerve blocks via various techniques that focus on the sympathetic nervous system at three main locations: the impar ganglion, the hypogastric plexus, and the lumbar sympathetic chain in L2 [13]. Neurolysis of the inferior hypogastric plexus has also been indicated in the treatment of pelvic and perineal pain caused by cancer [5]. A fluoroscopy-guided trans-sacral approach to the inferior hypogastric plexus showed a reduction in pain scores from a mean of 7.22 ± 1.31 to 4.06 ± 1.73 one week after the procedure [14]. Combinations of techniques, such as the neurolytic block of both the superior hypogastric plexus and the ganglion impar, have been shown to be an effective technique in cases of cancer-related lower abdominal and pelvic pain [15-17].

The superior hypogastric plexus is a branching network of intersecting nerves that lies retroperitoneally, spanning from the lower third of the fifth lumbar vertebral body to the upper third of the first sacral vertebral body. It consists of afferent pain fibers from pelvic structures, such as the bladder, urethra, uterus, perineum, prostate, penis, rectum, and descending colon [15]. The superior hypogastric plexus block (SHPB) is indicated for pelvic visceral pain and pelvic cancer pain. The classic technique of SHPB described by Plancarte et al. in 1990 was a fluoroscopy-guided, posterior, two-needle approach [18-20]. This classic technique was found to reduce pain in 70%-90% of patients experiencing pelvic pain secondary to cervical, prostate, and testicular cancer or radiation injury [18,19]. Another study found similar outcomes when performing the SHPB using the classic technique on 180 patients with visceral pelvic pain associated with malignancy (55% being cervical cancer and 11% with ovarian cancer), with a 55.5% pain reduction found at 33 months [18]. Another study utilizing the classic technique found pain reduction in 72% of patients suffering from gynecologic, colorectal, or genitourinary cancer (including 142 females with gynecological cancer and 17 males with prostate, colorectal, or bladder carcinomas) [21]. Other techniques for SHPB include transdiscal, posteromedian, posterior paravertebral, and CT-guided anterior and posterior approaches [5,8,18,20,22].

SHPB has also been indicated for general pain reduction with cases of noncancer-related, chronic pelvic pain caused by conditions such as endometriosis, pelvic inflammatory disease (PID), uterine fibroids, etc., with pain relief shown in 50%-70% of patients [19,23]. A CT-guided anterior unilateral approach has been indicated in two case reports. The first was used in a 43-year-old woman with the burning pain of the urethra after over two years of unrelieved pain using analgesics and antidepressants. The second case was a 68-year-old male with chronic burning and itching pain of the urethra and glans penis after unalleviated pain from anti-inflammatory drugs, tramadol, spasmolytics. In both cases, the patients reported significant pain relief six hours post-procedure [24].

Focusing on the management of intractable penile pain from non-cancer-related pathophysiology, literature has shown the use of various procedures. One case report showed the use of a pudendal nerve block via pulsed radiofrequency (RF) ablation in the treatment of pelvic nerve neuropathy. This procedure blocks the pudendal nerve signals via RF, which supplies both sensory and somatic sensations to the penis [25]. Studies have also shown the use of ultrasound-guided dorsal penile nerve blocks (DPNB) to treat penile pain, most commonly due to circumcisions or trauma. One case report shows the use of DPNB (ultrasound-guided with 4% phenol) in a patient with chronic pain due to penile calciphylaxis, resulting in immediate pain relief [26]. This procedure has also shown to be effective in a case report of malignant priapism, a rare and painful condition usually managed with palliative pain treatment [27].

A bilateral superior hypogastric plexus neurolysis is a time efficient and minimally invasive procedure targeted to alleviate pelvic pain via the interruption of nerve signal transmission of the plexus bilaterally. The authors believe this procedure to be an effective and superior alternative for the management of intractable penile pain, particularly due to neoplastic-related and radiation-induced injury due to its efficacy and safety, as well as the typically bilateral distribution of cancer-related pain [21]. On the contrary, the use of the currently indicated treatment algorithm of the analgesic ladder to treat intractable penile pain may produce many adverse effects. Examples include gastrointestinal system irritation, nephritis, hypersensitivity, sedation, etc. Additionally, the use of opiates comes with a significant risk of abuse, tolerance, and dependence thus leading to addiction and further worsening an already existing opioid crisis [4]. Furthermore, other current forms of management, such as the ultrasound-guided DPNB with phenol discussed above, can produce adverse effects due to the ability of phenol to potentially cause local tissue damage, dysesthesia, prolonged motor paralysis, bowel and bladder dysfunction, renal toxicity, and cardiovascular depression [26]. While SHPB has been shown to be an effective procedure in cases of pelvic and perineal visceral pain attributed to the following regions: appendix, colon, cervical, endometrium, stomach, intestine, liposarcoma, breast, bone marrow, ovarian, pancreas, prostate, lung, rectum, testicles, vagina, bladder, and gallbladder, the authors wish to raise awareness for the use of a bilateral approach specifically in patients suffering from intractable penile pain, due to both cancer- and non-cancer- related pathophysiology.

## Conclusions

This case presentation depicts the successful utilization of a bilateral superior hypogastric plexus neurolysis as an effective treatment option in the management of intractable neoplasm-related penile pain attributed to both radiation-induced injury in the treatment of malignant neoplasm and penile pain secondary to metastatic prostate cancer to the penis. As a currently under-utilized treatment option in the management of intractable penile pain, this case presentation acts to increase awareness of its potential use, therefore reducing the need for analgesics and the associated burdens, as well as improving patient palliation. Furthermore, this case offers evidence supporting the encouragement of its use in the general management of intractable penile pain due to other pathophysiology.

## References

[1] Delavierre D, Rigaud J, Sibert L, Labat JJ (2010). Approche symptomatique des douleurs péniennes chroniques [Symptomatic approach to chronic penile pain]. Prog Urol.

[2] Davaro FM, Weinstein D, Siddiqui SA, Hamilton ZA (2020). A lack of palliative therapy use in patients with advanced penile cancer. J Palliat Care.

[3] Kongsgaard U, Kaasa S, Dale O (2005). Palliative Treatment of Cancer-Related Pain.

[4] Rocha A, Plancarte R, Nataren RGR, Carrera IHS, Pacheco VALR, Hernandez-Porras BC (2020). Effectiveness of superior hypogastric plexus neurolysis for pelvic cancer pain. Pain Physician.

[5] Fronk B, Doulatram GR (2018). Hypogastric plexus blocks. Essentials of Interventional Techniques in Managing Chronic Pain.

[6] Sayed D, Grace PD, Wetherington BH (2019). Celiac plexus block and superior hypogastric plexus block. Deer's Treatment of Pain.

[7] Orhan ME, Bilgin F, Ergin A, Dere K, Güzeldemir ME (2008). Pain treatment practice according to the WHO analgesic ladder in cancer patients: eight years experience of a single center [Article in Turkish]. Agri.

[8] Bhatnagar S, Gupta M (2015). Evidence-based clinical practice guidelines for interventional pain management in cancer pain. Indian J Palliat Care.

[9] Vargas-Schaffer G (2010). Is the WHO analgesic ladder still valid? Twenty-four years of experience. Can Fam Physician.

[10] Laufenberg-Feldmann R, Schwab R, Rolke R, Weber M (2012). Cancer pain in palliative medicine [Article in German]. Anaesthesist.

[11] Ghoneim AA, Mansour SM (2014). Comparative study between computed tomography guided superior hypogastric plexus block and the classic posterior approach: a prospective randomized study. Saudi J Anaesth.

[12] Koizuka S, Nakajima K, Mieda R (2014). CT-guided nerve block: a review of the features of CT fluoroscopic guidance for nerve blocks. J Anesth.

[13] Hayek SM, Shah A (2014). Nerve blocks for chronic pain. Neurosurg Clin N Am.

[14] Eberlin KR, Pickrell BB, Hamaguchi R, Hagan RR (2021). Reset neurectomy for cutaneous nerve injuries. Plast Reconstr Surg Glob Open.

[15] Johnston S, Kraus J, Tutton S, Symanski J (2020). Ultrasound-guided diagnostic deep peroneal nerve blocks prior to potential neurectomy: a retrospective review. Skeletal Radiol.

[16] Rigaud J, Delavierre D, Sibert L, Labat JJ (2010). Sympathetic nerve block in the management of chronic pelvic and perineal pain. [Article in French]. Prog Urol.

[17] Plancarte R, Amescua C, Patt RB, Aldrete JA (1990). Superior hypogastric plexus block for pelvic cancer pain. Anesthesiology.

[18] Urits I, Schwartz R, Herman J (2021). A comprehensive update of the superior hypogastric block for the management of chronic pelvic pain. Curr Pain Headache Rep.

[19] de Leon-Casasola OA, Kent E, Lema MJ (1993). Neurolytic superior hypogastric plexus block for chronic pelvic pain associated with cancer. Pain.

[20] Mohamed SA, Ahmed DG, Mohamad MF (2013). Chemical neurolysis of the inferior hypogastric plexus for the treatment of cancer-related pelvic and perineal pain. Pain Res Manag.

[21] 21] Ahmed DG, Mohamed MF, Mohamed SA-E (2021). Superior hypogastric plexus combined with ganglion impar neurolytic blocks for pelvic and/or perineal cancer pain relief. Pain physician.

[22] Mercadante S (2019). The combination of superior hypogastric plexus block and the block of the ganglium impair in a patient with abdominal and perineal pain poorly responsive to opioids. J Pain Symptom Manage.

[23] Hasoon J, Urits I, Orhurhu V, Viswanath O, Aner M (2020). Role of interventional pain management in patients with chronic pelvic pain. Proc (Bayl Univ Med Cent).

[24] Michalek P, Dutka J (2005). Computed tomography-guided anterior approach to the superior hypogastric plexus for noncancer pelvic pain: a report of two cases. Clin J Pain.

[25] Bui C, Pangarkar S, Zeitlin SI (2013). Relief of urinary urgency, hesitancy, and male pelvic pain with pulse radiofrequency ablation of the pudendal nerve: a case presentation. Case Rep Urol.

[26] Mach S, Collie MA, Pesce MB (2021). Palliative nerve block for penile calciphylaxis: a case report on ultrasound-guided phenol neurolysis. A A Pract.

[27] Carvajal G, Rocha A (2019). Ultrasound-guided dorsal penile neurolysis for malignant priapism pain management. J Pain Symptom Manage.

